# Construction, Expression, and Characterization of Thymosin Alpha 1 Tandem Repeats in *Escherichia coli*

**DOI:** 10.1155/2013/720285

**Published:** 2013-02-28

**Authors:** Xiao-Chang Xue, Zhen Yan, Wei-Na Li, Meng Li, Xin Qin, Cun Zhang, Qiang Hao, Zeng-Lu Wang, Ning Zhao, Wei Zhang, Ying-Qi Zhang

**Affiliations:** State Key Laboratory of Cancer Biology, Department of Biopharmaceutics, School of Pharmacy, Fourth Military Medical University, Xi'an 710032, China

## Abstract

Thymosin alpha 1 (T**α**1), which is composed of 28 amino acids, has been commercialized worldwide for its immune-modulatory and antitumor effects. T**α**1 can stimulate T cell proliferation and differentiation from bone marrow stem cells, augment cell-mediated immune responses, and regulate homeostasis of immune system. In this study, we developed a novel strategy to produce T**α**1 concatemer (T**α**1*③*) in *Escherichia coli* and compared its activity with chemically synthesized T**α**1. Results showed that T**α**1*③* can more effectively stimulate T cell proliferation and significantly upregulate IL-2 receptor expression. We concluded that the expression system for T**α**1 concatemer was constructed successfully, which could serve as an efficient tool for the production of large quantities of the active protein.

## 1. Introduction

The tandem repeats of proteins and peptides are studied widely and formidable progress has been made in this field. It was reported that tandem amino acid repeats have many functions of stabilizing proteins [1], maintaining conformation [2], elevating activity, and increasing half-life of proteins in blood or tissues. Frasch and colleagues suggested that tandem repeats present in *Trypanosoma cruzi* transsialidase stabilized the catalytic activity. In addition, repeats present on *T*. *cruzi* shed proteins increased trans-sialidase half-life in blood from 7 to almost 35 h [3]. Some proteins that contain tandemly repeated sequences play important roles in cell membrane skeleton system [4, 5].

Thymosin alpha1 (T*α*1) is a heat-stable, acidic polypeptide composed of 28 amino acid residues blocked at the N-terminus by an acetyl group [6, 7]. It is an immune modifier that has been shown to trigger lymphocytes maturation, augment T cell function, induce T-cell differentiation, and promote reconstitution of immune defects. All these findings showed that T*α*1 could be a useful restorative therapeutic agent in the treatment of immunodeficiency diseases and immunosuppressed conditions [8–10].

In this study, T*α*1*③* which was composed of three repeated copies of T*α*1 was fuse-expressed with thioredoxin (trx) in *E. coli* TOP10 strain and purified by heat treatment and Q-Sepharose Fast Flow ion-exchange chromatography. Then, T*α*1*③* was released by treatment with 0.5 M Cyanogen bromide (CNBr) and purified by SP-Sepharose Fast Flow chromatography. In our strategy, trx acts as a chaperon to help T*α*1*③* folding and CNBr treatment removed any exogenous amino acid (such as Met at the N-terminus for translation start) from T*α*1*③* molecule. So we can get the “natural” T*α*1*③*. Finally, the biological activity of T*α*1*③* on T lymphocyte proliferation and IL-2R expression was assessed.

## 2. Materials and Methods

### 2.1. Materials

 Restriction enzymes, Taq DNA polymerase, and T4 DNA ligase were purchased from TaKaRa. Expression vector pThioHisA and *E. coli* strain TOP10 (F-mcrAΔ(mrr-hsd RMS-mcrBC) *φ*80 lacZΔM15 ΔlacX74 recA1 araΔ139Δ(ara-leu)7697 galU galK rpsL (Strr)endA1 nupG) were from Invitrogen. DNA fragments were synthesized in BIOASIA. Synthetic T*α*1 (ZADAXIN) was from Sciclone Pharmaceuticals, USA. The anti-T*α*1 antibody (ab55635) was purchased from Abcam and FITC-anti-IL-2R*β* (18344D 554452) was from BD Pharmingen.

### 2.2. T*α*1*③* Gene Amplification

**Figure 1:**
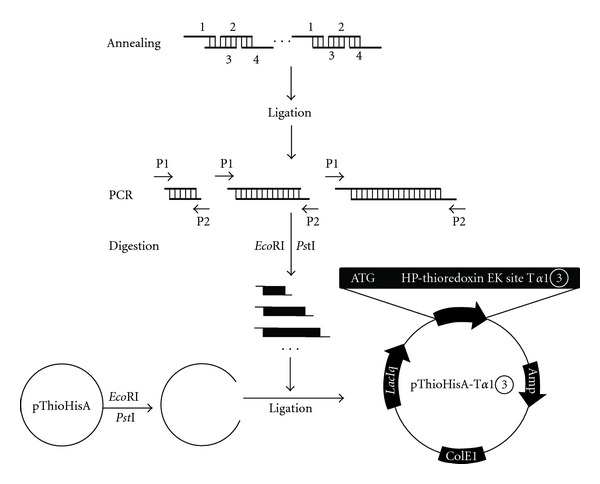
Schematic diagram shows the strategy for constructing T*α*1 concatemers. The Arabic numerals 1 to 4 are sequences 1 to 4 split from T*α*1 described previously for concatemers assembly. P1 and P2 are forward and reverse primers for PCR.

T*α*1*③* gene was cloned by gene synthesis and PCR (Figure 1). The forward primer (with an introduced *Eco*R I site) was p1: 5′-GGAATTCATGTCTGATGCAGCCGTGGAC ACCAGCAGCG-3′ and the reverse primer (with an introduced *Pst* I site) was p2: 5′-GCACTGCAGTCAGTTCTGGGCCTCCTCCACCACCT-3′. The template for PCR was annealing products of 4 synthesized fragments listed in Table 1. PCR product was cloned into pGEM-3Zf plasmid to obtain vector pGEM-T*α*1*③* for identification.

### 2.3. Construction of Expression Vector pThioHisA-T*α*1*③*

The vector pGEM-T*α*1*③* was digested with *Eco*R I and *Pst *I and cloned into expression vector pThioHisA digested with the same enzymes. The candidate plasmid pThioHisA-T*α*1*③* was then confirmed by restriction enzymes digestion and DNA sequencing. 

### 2.4. Expression of the Fusion Protein

The plasmid pThioHisA-T*α*1*③* was transformed into *E. coli* TOP10 strain. A single colony was inoculated into 10 mL Luria-Bertani (LB) medium supplemented with ampicillin (100 *μ*g/mL) and grown at 200 rpm and 37°C overnight. Then it was inoculated into 300 mL fresh LB medium in a 500 mL shake flask and cultured until the OD600 reached 0.5. Trx-T*α*1*③* expression was induced by 1 mM IPTG (final concentration) for 4 h. Large scale fed-batch culture was performed in a 5-L fermentor as previously described [11].

### 2.5. Purification of T*α*1*③*

Cell pellet was suspended in 20 mM Tris/HCl buffer (pH 8.0) in proportion of 200 g/L and disrupted by sonication. Then, the lysate was incubated at 80°C for 10 min (shaken once every 2-3 min) and cooled quickly. Samples were centrifuged at 12 000 g for 20 min and the supernatant was loaded onto a Q-Sepharose Fast Flow chromatography column and eluted with linear NaCl gradient. The purified Trx-T*α*1*③* was then cleaved by CNBr (0.5 M) in 70% formic acid for 24 h. The cleavage reaction was stopped by addition of ten times amount of H_2_O [12] and T*α*1*③* was purified by SP-Sepharose Fast Flow chromatography. Purified T*α*1*③* was dialyzed against PBS for later use. 

### 2.6. Western-Blot Analysis

Proteins were transferred to nitrocellulose membranes (0.22 *μ*m, Invitrogen) after SDS-PAGE using a Bio-Rad Semi-Dry electrophoretic cell. Western blot analyses were carried out using a T*α*1 specific antibody and followed by a phosphatase-conjugated goat anti-mouse IgG (Boster, China). Western Blue Stabilized Substrate (Promega) for alkaline phosphatase was used for visualization. 

### 2.7. Biological Activity Assay

The proliferation response of splenocytes was determined by MTT assay. Spleens from C57BL6 mice were dispersed through nylon mesh to generate a single-cell suspension. Then lymphocytes were separated by EZ-Sep 1× Lymphocyte Separation Medium (DKW33-R0100, Dakewe Biotech Company, China) and suspended at 4 × 10^6^/mL in RPMI 1640 media. For proliferation assay, cells were seeded in 96-well plates (4 × 10^5^/well) and cultured in the presence of 2.5 *μ*g/mL concanavalin A (ConA) at 37°C in 5% CO_2_ in humid air. Six h later, 90 *μ*L of T*α*1*③* diluted with RPMI 1640 media was added to all but the control wells. The synthetic T*α*1 and media were used as positive and negative controls. After 66 h incubation, 20 *μ*L of MTT (0.5 mg/mL) solution was added and the plates were centrifuged (2000 rpm, 25°C, 10 min) 4 h later. Supernatants were discarded, and 100 *μ*L of DMSO was added. After incubated at room temperature for 10 min, the solubilized reduced MTT was measured at 570 nm using a Bio-Rad plate reader and the optical densities were used for calculate growth rate with the formula

(1) $$\begin{array}{c} \text{Growth}\;\text{rate}\;\left(\%\right)=\frac{\;\text{OD}\;\text{sample}}{\text{OD}\;\text{control}}\times 100\%. \end{array}$$

To evaluate the effect of T*α*1*③* on the expression level of IL-2R on T lymphocytes, cells were isolated as before and cultured in the presence of ConA and T*α*1*③*. The synthetic T*α*1 and a recombinant T*α*1 monomer prepared in our lab were used as positive controls. Cells were collected and stained 48 h later according to standard protocol. In brief, 5 × 10^5^ cells were washed with PBS and stainedin “FACS buffer” (PBS with 0.1% sodium azide, 2% FBS, and 1 *μ*M EDTA) with FITC-anti-mIL-2R*β* for 10 min at room temperature. After washing, cells were fixed for 30 minutes on ice with 4% paraformaldehyde and analyzed on a FACSCalibur flow cytometer (BD Biosciences). 

## 3. Results and Discussion

### 3.1. T*α*1*③* Gene Cloning

 Although synthetic T*α*1 has been successfully applied in clinical trials for immunodeficiency diseases therapy, the high costs is still a hard nut to crack. Fortunately, molecular biology techniques allowed us to produce recombinant T*α*1 in *E. coli*. Considering that T*α*1 is too small to be directly expressed in *E. coli*, it was usually assembled as concatemers. But some exogenous amino acid residues such as His6 tag or methionine (Met) introduced by the initiation codon AUG usually affects the effect of concatemers [13]. 

In order to produce the real “natural” concatemers of T*α*1, we put forward a new strategy as showed in Figure 1. By this strategy, we obtained a series of T*α*1 concatemers in which T*α*1*③* that was assembled by three repeated copies of T*α*1 gene owned highest proportion. After cloning into pGEM-3Zf vector, the gene was proven by enzyme digestion and DNA sequencing. The sequence of T*α*1*③* gene was consistent with our design as follows: 5′-atgagcgacgccgccgtggacaccagcagcgagatcaccaccaaggaccggaaggagaagaaggaggtggtggaggaggccgagaacagcgacgccgccgtggacaccagcagcgagatcaccaccaaggaccggaaggagaagaaggaggtggtggaggaggccgagaacagcgacgccgccgtggacaccagcagcgagatcaccaccaaggaccggaaggagaagaaggaggtggtggaggaggccgagaactga-3′. 

### 3.2. Expression of Recombinant Fusion Protein

**Figure 2:**
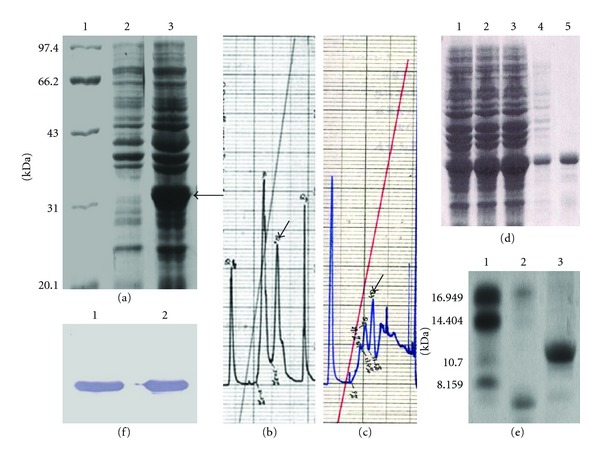
Expression, purification, and identification of T*α*1*③*. (a) Expression of trx-T*α*1*③* in TOP10. Lane 1: protein marker; lane 2: total bacterial proteins of pThioHisA-T*α*1*③*/TOP10 without induction; lane 3: total bacterial protein with IPTG induction. (b) Chromatogram of Q-Sepharose Fast Flow chromatography for purification of trx-T*α*1*③*. The arrow indicated trx-T*α*1*③*. (c) Chromatogram of SP-Sepharose Fast Flow chromatography for purification of T*α*1*③*. The arrow indicated T*α*1*③*. (d) SDS-PAGE analysis of trx-T*α*1*③* purification. Lane 1–3: total proteins of pThioHisA-T*α*1*③*/TOP10 after IPTG induction (1 h, 3 h, 5 h); lane 4: supernatant of lysate heated at 80°C for 10 min; lane 5: purified trx-T*α*1*③*. (e) Tricine-SDS-PAGE analysis of T*α*1*③* purification. Lane 1: standard peptide marker; lane 2: cleavage products without T*α*1*③*; lane 3: purified T*α*1*③*. (f) Western-blot analysis of trx-T*α*1*③*. Lane 1: total bacterial proteins after IPTG induction; lane 2: purified trx-T*α*1*③*.

Both SDS-PAGE (Figure 2(a)) and Western blot (Figure 2(f)) analyses of the induced supernatant from pThioHisA-T*α*1*③*/TOP10 showed that a new 31 kDa protein which can be specifically recognized by T*α*1 antibody was produced. It suggested that trx-T*α*1*③* was successfully expressed. Trx was used as a chaperon to guarantee the correct folding of T*α*1*③* and trx-T*α*1*③* was expressed as a soluble fusion protein. 

### 3.3. Purification of T*α*1*③*

Both trx and T*α*1 are heat-stable proteins, so trx-T*α*1*③* was easily purified by one-step Q-Sepharose Fast Flow chromatography after the lysate of recombinant bacterial cells was heated at 80°C for 10 min (Figures 2(b) and 2(d)). Then, the purified trx-T*α*1*③* was cleaved by CNBr, and T*α*1*③* was purified by SP-Sepharose Fast Flow chromatography (Figures 2(c) and 2(e)). 2L-Tricine-SDS-PAGE [14] and HPLC analyses were used to identify the purity of T*α*1*③*. CNBr treatment was utilized here to remove the redundant Met from the N-terminus of T*α*1*③* to obtain the real “natural” molecule. 

### 3.4. Biological Activity of  T*α*1*③*

We expected that the tandem repeats could obtain stronger activity through elongating the half-life of T*α*1 and simulating polymerization of monomer molecules and thereafter triggering the polymerization and activation of receptors which was usually used by molecules to gain function. 

**Figure 3:**
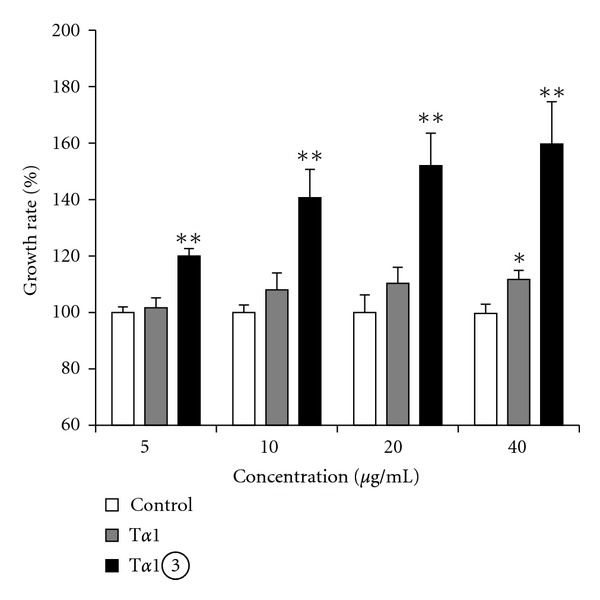
T*α*1*③* stimulated proliferation of mouse spleen lymphocytes. T lymphocytes from B6 mice spleen were treated with ConA (control) or ConA plus T*α*1*③* or synthetic T*α*1. Cell proliferation was determined by the MTT viability assay. The assays were repeated in triplicate. (*P < 0.05 compared with control group. **P < 0.05 compared with T*α*1 group.)

To examine the effect of T*α*1*③* on stimulating the proliferation of splenic lymphocytes, we compared the proliferation ratio of mice lymphocytes treated with synthetic T*α*1 ZADAXIN and T*α*1*③*. MTT assay results showed that 40 *μ*g/mL synthetic T*α*1 could induce significant proliferation of lymphocytes compared to the control (*P* < 0.05), whereas 5 *μ*g/mL T*α*1*③* could induce significant proliferation (*P* < 0.05). Furthermore, the effect of 10 *μ*g/mL T*α*1*③* was stronger than that of 40 *μ*g/mL synthetic T*α*1 (Figure 3). 

**Figure 4:**
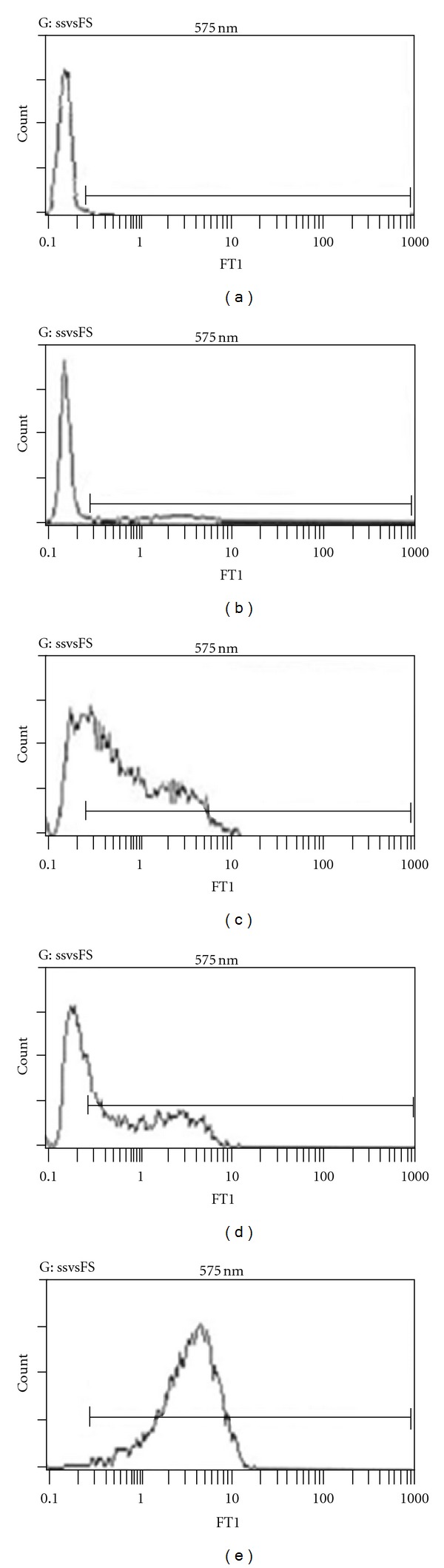
Effect on upregulation of IL-2R expression level on T cell surface of T*α*1*③*. T lymphocytes from B6 mice spleen were cultured in the presence of ConA (b) or ConA plus standard synthesized T*α*1 (c), recombinant T*α*1 (d), and recombinant T*α*1*③* (e), respectively. (a) The unstrained control. Data are representative of three experiments.

In addition, the upregulation of IL-2R on lymphocytes by ZADAXIN purified recombinant T*α*1 monomer and T*α*1*③* was compared. Results showed that when costimulated with ConA, IL-2R expression level on T cell was upregulated by all these three molecules and T*α*1*③* obtained strongest effect (Figure 4). 

## 4. Conclusions 

Trx-T*α*1*③* was expressed in *E. coli* as a soluble form and the real “natural” T*α*1*③* was conveniently purified by heat treatment and ion-exchange chromatography. As expected, the bioactivity of T*α*1*③* was stronger than that of synthetic T*α*1. Lower dose (5 *μ*g/mL) of T*α*1*③* apparently stimulated the proliferation of T lymphocytes compared with that of ZADAXIN (40 *μ*g/mL). In addition, T*α*1*③* significantly upregulated IL-2R on T cell, which is very important for T cell activation and proliferation *in vivo*. The detailed mechanism for stronger effect of T*α*1*③* and the pharmacokinetics of different tandem repeats are still under investigation. 

## Figures and Tables

**Table 1:** Nucleotide sequence of DNA fragments split from T*α*1 for concatemers assembly.

Number	nucleotide sequence
1	GACACCAGCAGCGAGATCACCACCAAGGACC GGAAGGAGAAGAAGGAGGTGGTG
2	GAGGAGGCCGAGAACAGCGACGCCGCCGTG
3	CTTGGTGGTGATCTCGCTGCTGGTGTCCACGG CGGCGTCGCT
4	GTTCTCGGCCTCCTCCACCACCTCCTTCTTCTCC TTCAGGTC
